# Valence isomerization of cyclohepta-1,3,5-triene and its heteroelement analogues

**DOI:** 10.3762/bjoc.7.201

**Published:** 2011-12-21

**Authors:** Helen Jansen, J Chris Slootweg, Koop Lammertsma

**Affiliations:** 1 Department of Chemistry and Pharmaceutical Sciences, VU University Amsterdam, De Boelelaan 1083, 1081 HV, Amsterdam, The Netherlands

**Keywords:** aromaticity, cycloheptatriene, heteroatom, valence isomerization

## Abstract

The valence isomerization of the all-carbon and heteroelement analogues of cyclohepta-1,3,5-triene into the corresponding bicyclo[4.1.0]hepta-2,4-dienes is reviewed to show the impact of the heteroatom on the stability of both valence isomers. The focus is on the parent systems and their synthetic applications.

## Introduction

**Scheme 1:**
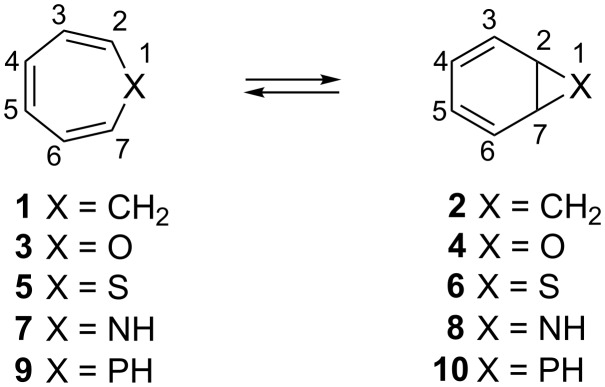
Valence isomerization of cyclohepta-1,3,5-triene (**1**) and its heteroelement analogues.

The valence isomerization of cyclohepta-1,3,5-triene (**1**) into bicyclo[4.1.0]hepta-2,4-diene (**2**) has captured the attention of chemists for over five decades [1–2]. This interest extended to the heterocyclic analogues **3**–**8**, bearing one oxygen, sulfur or nitrogen atom, after the discovery of their biological importance (Scheme 1) [3–4]. The phosphane analogues **9** and **10** received far less attention, with their applicability as a phosphinidene (R–P) precursor being the most notable use [5–9].

Reviewing the influence of the heteroatom on the cycloheptatriene–norcaradiene valence isomerization necessitates a brief overview of the parent all-carbon system. This section is followed by one in which experimental data on the oxepine, thiepine, 1*H*-azepine, and 1*H*-phosphepine valence isomerizations are compared with those obtained by theoretical calculations. Computational methods have the advantage that they enable reliable insight into the reaction energies and aromatic features of the parent isomers. In this brief review, only selected examples of substituted heteropines and their syntheses are given.

## Review

### Cycloheptatriene valence isomerization

**Scheme 2:**
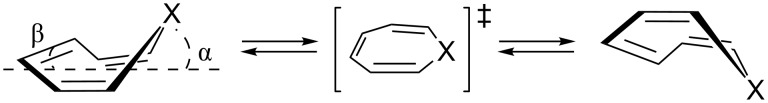
Conformational ring inversions.

Cyclohepta-1,3,5-triene (**1**), first isolated in 1883 [10], has a boat-shaped conformation as determined by electron diffraction [11] and microwave studies of the parent [12] and by an X-ray structure analysis of the derivative thujic acid [13–14]. These methods gave inconsistent α and β tilt angles (see Scheme 2 for a description of the bow (α) and stern (β) tilt angles) with those determined by electron diffraction standing out. Theoretical calculations at the B3LYP/6-311+G(d,p) level gave α and β angles of 52.9° and 25.4°, respectively [15–17], which are in reasonable harmony with those of the microwave and X-ray studies. Low temperature ^1^H NMR measurements showed that the slightly homoaromatic boat conformation is prone to undergo a degenerate ring flip via an antiaromatic *C*_2_*_v_* transition with a free energy barrier of 5.7 kcal·mol^−1^ in CBrF_3_ [18] and 6.3 kcal·mol^−1^ in CF_2_Cl_2_ [19–21].

Cycloheptatriene is in equilibrium with bicyclo[4.1.0]hepta-2,4-diene (**2**) by means of a Woodward–Hoffmann symmetry-allowed disrotatory ring closure [22–23]. Although the equilibrium strongly favours the seven-membered ring, the presence of small quantities of the bicyclic isomer **2** was inferred by Diels–Alder trapping reactions [24]. In 1981, Ruben was the first to observe norcaradiene (**2**) directly, by employing low-temperature photolysis, and an activation barrier of 11 ± 2 kcal·mol^−1^ was determined for the formation of **2** from **1**, with the product being 4 kcal·mol^−1^ less stable [25]. Strong electron-withdrawing groups at the methylene bridge influence the **1**–**2** equilibrium in favour of the norcaradiene isomer, as is the case for the thermally stable 7,7-dicyano-derivative [26–27]. At the B3LYP/6-311+G(d,p) level the geometry of the parent was shown to have a straighter bow (α = 65.8°) and flatter stern (β = 18.9°) as compared to cyclohepta-1,3,5-triene [14].

**Scheme 3:**
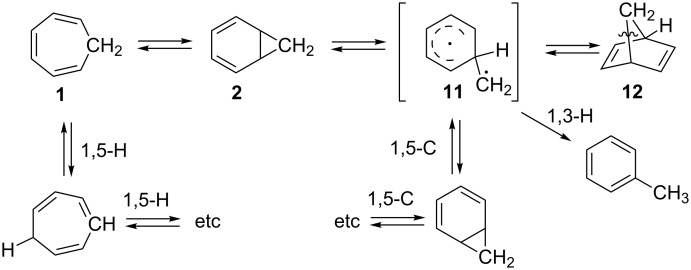
Rearrangements of the parent cycloheptatriene **1** and norcaradiene **2**.

Besides the **1**–**2** interconversion, the C_7_H_8_ system is rich in rearrangements (Scheme 3). In 1957, Woods found that bicyclo[2.2.1]hepta-2,5-diene (**12**) converts to cycloheptatriene (**1**), which was postulated to proceed via diradical **11** and norcaradiene (**2**) [28]. Instead, pyrolysis of **1** yielded toluene, presumably through a [1,3]-H shift of the diradical [29]. Norcaradiene (**2**) can also undergo a [1,5]-carbon circumambulatory rearrangement (“walk”), as was discovered by Berson and Willcott in 1965 [30–31]. Although, this process should proceed with retention of the configuration according to the symmetry conservation rules, studies of chiral substituted cycloheptatrienes showed a preference for the “forbidden” path with inversion of configuration [32–35]. Finally, a suprafacial [1,5]-hydrogen shift with an activation energy of approximately 31 kcal·mol^−1^ was unveiled by a high-temperature NMR study (100–140 °C) of hydrogen isotopomers of cycloheptatriene (Scheme 3) [36–38].

### Valence isomerization of heteropines

Determining the conformations of the heteropines has been more of a challenge. Only the parent oxepine (**3**) is isolable at room temperature. NMR spectroscopy indicated a boat-shape structure with alternating C=C bonds for **3** [39–40], which was supported by single-crystal X-ray structure analyses of simple derivatives [41]. Table 1 also summarizes the relative energies obtained by high-level theoretical calculations for the parent heteropines and the corresponding bicyclic norcaradienes, and the barriers for their interconversion and ring inversion.

**Table 1:** Relative energies (in kcal·mol^−1^) of the norcaradienes (NCD) **2** (C), **4** (O), **6** (S), **8** (N), **10** (P), the cycloheptatrienes (CHT) **1** (C), **3** (O), **5** (S), **7** (N), **9** (P), their interconversion barriers, and the barriers for ring inversion of the monocycles.

	NCD	TS	CHT	TS_inv_	Method^a^	Ref

C (**1**,**2**)	4	11^b^	0.0	~6	Exp.	[17–18,24]
O (**3**,**4**)	0.0	9.1^c^	1.7	–	NMR	[38–39]
	0.0	7.0	0.1	3.5	QCISD(T)/6-31G(d)	[40]
S (**5**,**6**)	0.0	20.5^c^	7.0	7.3	QCISD(T)/6-31G(d)	[40]
N (**7**,**8**)	7.9	11.4^b^	0.0	~3	B3LYP/6-31G(d)	[41–42,44]
P (**9**,**10**)	0.0	15.7	2.5	5.2	B3LYP/6-311+G(d,p)	[7,45]

^a^Gibbs free energies for the experimental data (first two entries) and enthalpies for the computational data. ^b^Equilibrium from CHT to NCD. ^c^Equilibrium from NCD to CHT.

Although the boat form prevails for the monocyclic heteropines **1**, **3** and **5**, Cremer et al. showed that this represents an incomplete picture [43–44]. In fact, they are “perturbed” boats with at least 22% chair character, leading to an almost similar boat puckering for all. From the racemization of substituted benzene oxides (Scheme 2), the oxepine ring inversion barrier was estimated at 6.5 kcal·mol^−1^ at 135 K [45–46], which is similar to the 3.5 kcal·mol^−1^ calculated for the parent oxepine (**3**) at the QCISD(T)/6-31G(d) level [40]. The calculated barrier of 8.3 kcal·mol^−1^ for thiepine (**5**) is nearly twice as large, possibly due to the higher antiaromatic destabilization of the flattened thiepine ring [40], but the interconversion of the boat forms of azepine and phosphepine are about equally favourable, requiring 3.0 [41] and 5.2 kcal·mol^−1^ [7,47], respectively.

**Figure 1:**
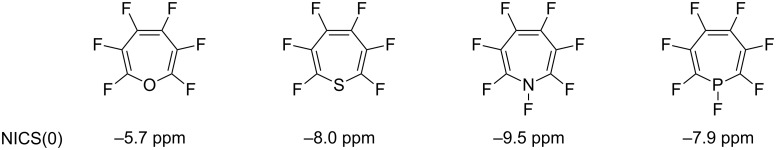
NICS(0) values of fluorinated heteropines.

A question related to the valence isomerization is whether aromatic properties can be ascribed to the heteropines. Indeed, the monocyclic boat-shaped heteropines exhibit homoaromatic features by conjugative interaction of the triene unit through 1,6-overlap of 2p π-orbitals [41], as is the case for cyclohepta-1,3,5-triene (**1**) [19–20]. Through the use of nucleus-independent chemical shifts (NICS(1)) [48], it was shown that thiepine (−2.3 ppm) [49] and phenyl phosphepine (−4.8 ppm) [7] display aromatic character when compared to the well-known 6π-electron Hückel-aromatic tropylium cation [46], which has a NICS(1) value of −8.2 ppm. Adding electronegative substituents enhances the effect, and fully aromatic systems are obtained after complete fluorination of the heteropines (Figure 1) [50]. In contrast, the flattened transition structures for ring inversion of thiepine and phosphepine are indeed highly antiaromatic planar 8π-electron systems, with positive NICS(1) values of 19.3 [47] and 6.4 ppm [7], respectively. The inherent instability of thiepine (**5**) has been attributed to this effect [51–52].

### Oxepine – benzene oxide

Oxepine (**3**) was isolated first by Vogel et al. using a double dehydrohalogenation of 1,2-dibromo-4,5-epoxycyclohexane [38,53], but is also accessible by epoxidation of Dewar benzene followed by photolytic or thermal ring expansion [54]. The molecular structure of the 2-*tert*-butoxycarbonyl oxepine showed a boat configuration with bow (α) and stern (β) fold angles of 56.5° and 26.0°, respectively [44], which differs little from the MP2/6-31G(d) geometry of the parent **3** (*C_s_* symmetry; α = 58.3°, β = 30.8°), illustrating that the substituent hardly influences the geometry [40]. Oxepine (**3**) is more curved than cyclohepta-1,3,5-triene (**1**; α = 52.9°, β = 25.4°; same level of theory) [14].

**Scheme 4:**
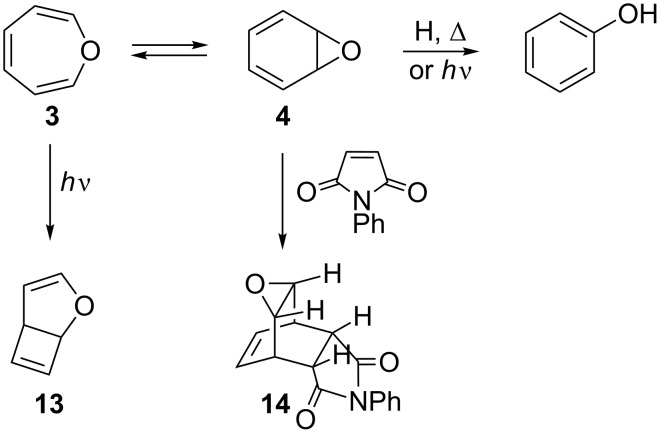
Reactivity of oxepine (**3**) and benzene oxide (**4**).

Using ^1^H NMR spectroscopy, Vogel and Günther determined that 7-oxa-bicyclo[4.1.0]hepta-2,4-diene (**4**, benzene oxide; Scheme 4) is 1.7 kcal·mol^−1^ more stable than monocyclic **3** in apolar solvents [38–39], with an activation barrier for the conversion of **3** to **4** of 7.2 kcal·mol^−1^. Calculations at the QCISD(T)/6-31G(d) level confirm the bicyclic form to be the most stable isomer, albeit with an energy difference of a mere 0.1 kcal·mol^−1^ and a barrier to interconversion of 9.1 kcal·mol^−1^ [40]. By changing to more polar solvents, the oxepine isomerization equilibrium shifts further toward benzene oxide (more positive Δ*G*), suggesting that benzene oxide has the larger dipole moment. Methyl substitution at the 2- and 7-positions reverses the stability order, rendering the oxepine as the energetically favoured isomer due to the destabilizing eclipsing of the two methyl groups in benzene oxide (**4**) [38,40,51]. Thus, in contrast to the cycloheptatriene–norcaradiene (**1**–**2**) pair, the equilibrium constant for oxepine (**3**) and bicyclic benzene oxide (**4**) varies widely with solvent polarity and to some extent with temperature and substituents, making it possible to work with solutions highly enriched with either one or the other isomer [38,55]. The facile **3**→**4** valence isomerization [56–58], pioneered by the synthesis of 1,2-naphthalene oxide by Vogel and Klärner [1,59–60], is of considerable interest as arene oxides are intermediates in the oxidative metabolism of aromatic substrates [61–64]. In addition, also photo-oxidation of benzene creates this isomeric pair [65–66].

Depicted in Scheme 4 are the most important reactions that the parent oxepine (**3**) and benzene oxide (**4**) can undergo. Irradiation of oxepine results in ring contraction yielding 2-oxabicyclo[2.3.0]hepta-3,6-diene (**13**) [38,51], while under thermal, photochemical or acidic conditions, the three-membered ring of bicyclic **4** opens, generating phenol [67–68], in analogy to the all-carbon norcaradiene (**2**), which gives toluene. In addition, **4** undergoes highly selective Diels–Alder reactions, such as with *N*-phenylmaleimide and dimethyl acetylenedicarboxylate, providing single *anti*-adducts (e.g., **14**; Scheme 4) [61,69]. Theoretical calculations on model structures showed the *anti* cycloaddition to be the kinetically controlled path and the *syn* addition the thermodynamically favoured one [40].

### Thiepine – benzene sulfide

The parent thiepine (**5**) is 7.0 kcal·mol^−1^ less stable than benzene sulfide (**6**). This energy difference is much larger than for the oxygen homologues, because three-membered rings accommodate sulfur better than oxygen [40]. Nonetheless, bicyclic **6** has never been isolated, probably due to the low activation barrier for sulfur extrusion [40,48,70], which occurs through a sequence of low-energy processes involving several sulfur-containing intermediates [71–72].

**Figure 2:**
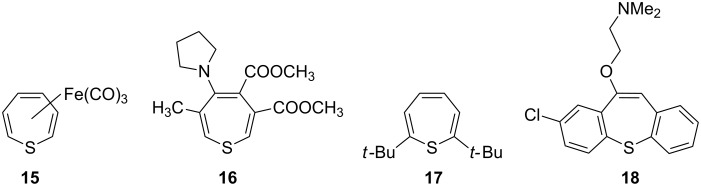
Stabilized thiepines **15**–**18**.

Thiepine (**5**) can be stabilized by Fe(CO)_3_ complexation (**15**; Figure 2) [73] or by decorating the seven-membered ring with substituents. The first isolated metal-free thiepine (**16**; Figure 2) was reported in 1974 by Reinhoudt and Kouwenhoven, who used electron-withdrawing groups to delocalize the π-electrons of the thiepine ring, but this species still eliminates sulfur at room temperature [74]. With the synthesis of the sterically shielded 2,7-di-*tert*-butylthiepine (**17**) (Figure 2), a relatively simple and thermally stable thiepine was obtained, allowing experimental studies of its chemical and physical properties [75]. A single-crystal X-ray analysis showed **17** to be less curved (α = 49.6° and β = 28.0°) [70] than the computed structure of cyclohepta-1,3,5-triene (**1**; α = 52.9°, β = 25.4°) [14]; The MP2/6-31G(d) optimized geometry of the parent thiepine (**5**) (α = 50.3° and β = 30.8°) [40] is similar to that of the molecular structure of **17** [76]. Benzannulation of the thiepine ring on both sides results in the thermally robust dibenzo[*b*,*f*]thiepines, which are of interest for their potent biological activity, illustrated by the psychosedative and antipsychotic properties of zotepine (**18**; Figure 2) [76–80].

### 1*H*-Azepine – benzene imine

The parent 1*H*-azepine (**7**) [81] was first generated in 1963 by Hafner, by the hydrolysis of ethyl-1*H*-azepine-*N*-carboxylate with potassium hydroxide and subsequent protonation [82]. Because 1*H*-azepine is highly unstable and rapidly undergoes a [1,3]-H shift to 3*H*-azepine, only an X-ray structure determination at −78 °C of an N-substituted derivative was reported by Vogel et al. 17 years later [83–85]. The molecular structure of *N*-(phenoxycarbonyl)azepine displays a rather shallow boat structure (α = 43.4° and β = 21.6°) [86], which is solely due to the N-substituent, as the CASSCF/3-21G optimized geometry showed a more curved β angle of 36.4° for the parent **7** [87].

**Scheme 5:**
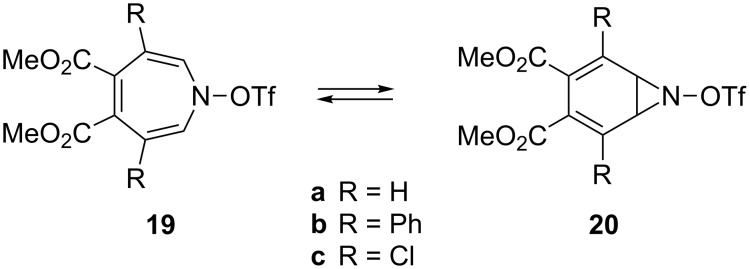
Valence isomerization of 1*H*-azepines.

Like the all-carbon analogues, the valence isomerization strongly favours the monocyclic form with an estimated preference of 7.9 kcal·mol^−1^ at the B3LYP/6-31G(d) level for the parent system (**7**→**8**) [42]. Also low temperature ^1^H and ^13^C NMR measurements on **19** display only small amounts of the bicyclic isomers **20** (Scheme 5) [79,88].

**Scheme 6:**
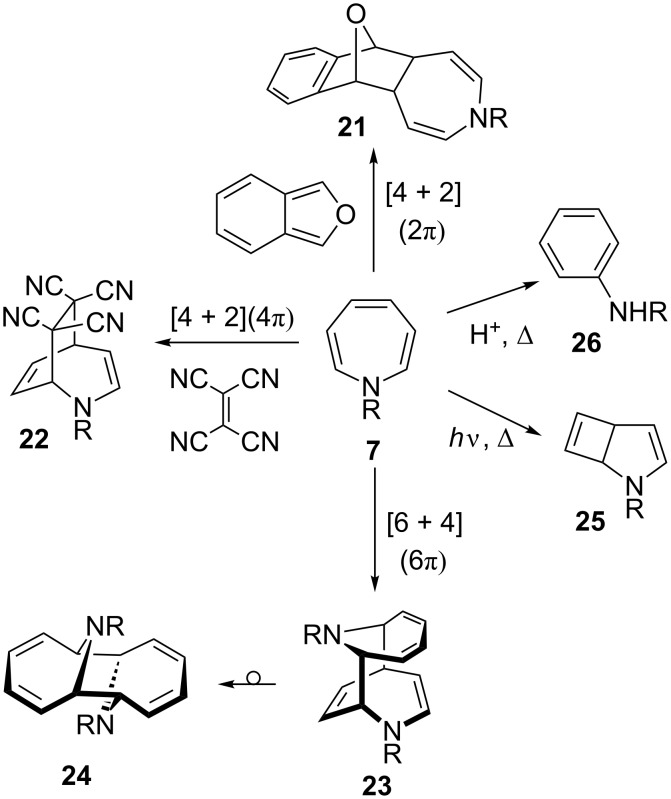
Reactivity of 1*H*-azepine.

The reluctance to form the bicyclic isomer dictates the reactivity of azepines, as they exhibit the characteristics of cyclic polyene chemistry, which is illustrated by the ability of the monocyclic isomer to undergo cycloadditions as a 2π (→**21**) [89], 4π (→**22**) [84,90], or 6π (→**23**, **24**) [91–92] component (Scheme 6). In addition, azepine (**7**) rearranges photochemically to bicyclic **25** [93], and in the presence of an acid yields aniline derivatives **26** [94] in analogy to the cycloheptatriene and oxepine [95].

**Figure 3:**
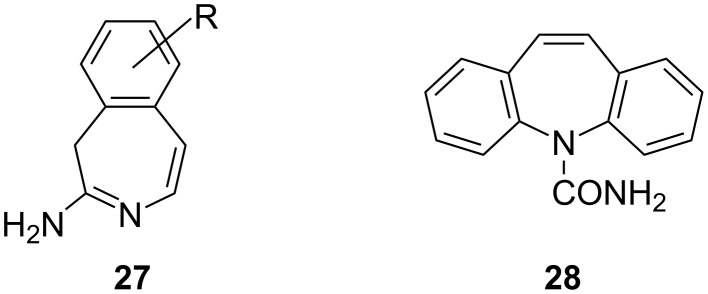
Benzannulated azepines **27** and **28**.

Like the thiepines, the benzannulated azepines have also received considerable attention due to their biological importance and pharmaceutical relevance [96]. For instance, 3*H*-3-benzazepin-2-amines **27** possess antihypertensive activity [97], and all tricyclic dibenzo[*b*,*f*]azepines (e.g., **28**; Figure 3) bearing a basic side chain affect the central nervous system [98].

### 1*H*-Phosphepine – benzene phosphane

**Figure 4:**
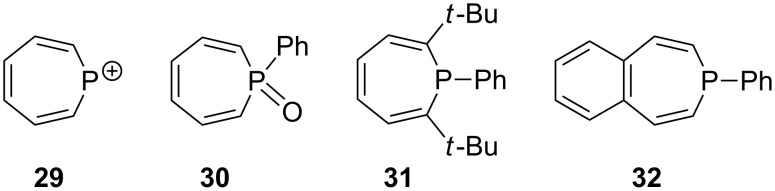
Reported phosphepines **29**–**32**.

**Scheme 7:**
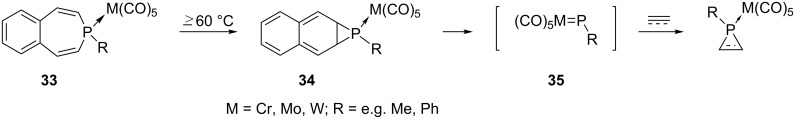
Phosphinidene generation from metal-complexed benzophosphepine **33**.

Although the parent 1*H*-phosphepine (**9**) and its 2.5 kcal·mol^−1^ more-stable valence isomer benzene phosphane (**10**) have never been isolated [45], there is evidence for the existence of the parent phosphatropylium ion (**29**; Figure 4), which was generated in the gas phase by collision activation between PI_3_ and benzene [99]. P-phenyl substitution stabilizes the phosphanorcaradiene (Δ*E* = 4.8 kcal·mol^−1^), but this species has also never been observed experimentally [7]. The thermal instability of the phosphepines and their valence isomers is due to the facile decomposition of the bicyclic phosphanorcaradiene (**10**) into benzene and phosphinidene R–P [100]. However, the 7-membered ring can be stabilized by phosphorus oxidation (**30**; see Figure 4) [95], the introduction of bulky substituents at the 2 and 7 positions (**31**) [101], or benzannulation (e.g., 3*H*-benzophosphepine, **32**) [7,102–107]. The single-crystal X-ray structure analysis of phenyl-substituted phosphepine **33** (Scheme 7) also showed a flattened-boat conformation (α = 40.5°, β = 28.2°) [5] compared to the metal-free parent structure (α = 48.3°, β = 27.8°), computed at the B3PW91/6-311+G(d,p) level [7].

Also for the phosphepine system [108], benzannulation leads to interesting targets. Namely, the thermal lability of the transition-metal-complexed 3*H*-benzophosphepine **33** was explored by Lammertsma et al. for the synthesis of a variety of organophosphorus compounds by means of [1 + 2] cycloadditions of the in situ generated singlet phosphinidene **35** with olefins or acetylenes (Scheme 7) [5–9]. This approach has even lead to the detection of the transient phosphinidene species by employing electrospray ionization tandem mass spectrometry (ESIMS/MS); its gas-phase reactivity perfectly matches the well-established solution-phase chemistry [109]. Using these phosphinidenes [110–111] led to the synthesis of unique P-ligands for catalysis [112–113] as well as to attractive building blocks for the creation of P-functionalized polymers [114–115].

## Conclusion

The valence isomerization of cyclohepta-1,3,5-triene into the parent norcaradiene, and of their corresponding heteroelement analogues, has been reviewed with a focus on the chemical and physical properties of these fascinating species. The presence of a heteroatom has an impact on the stability of the heteropines, of which to date only the parent oxepine has been isolated. The generation of these (transient) heterocycles allowed the development of a rich chemistry, which has been extensively explored using the full toolbox of physical organic chemistry.
